# An Experimental Study of Effects of Media Implication on Self-Report Symptoms Related With MP Use

**DOI:** 10.3389/fpubh.2020.00175

**Published:** 2020-05-13

**Authors:** Peng Gao, Fei-Zhou Zheng, Min-Di He, Min Li, Ping Deng, Zhou Zhou, Zheng-Ping Yu, Lei Zhang

**Affiliations:** ^1^Department of Occupational Health, Key Laboratory of Medical Protection for Electromagnetic Radiation, Ministry of Education, Third Military Medical University, Chongqing, China; ^2^General Hospital of Central Theater Command, Wuhan, China; ^3^Department of Environmental Medicine, Zhejiang University School of Medicine, Hangzhou, China; ^4^Department of Emergency Medicine, First Affiliated Hospital, Zhejiang University School of Medicine, Hangzhou, China

**Keywords:** media implication, health effect, mobile phone use, physical symptoms, depression

## Abstract

Along with gradually increases in mobile phone (MP) use, the mass media has played a vital role in informing the public regarding the potential health hazards of MP use. These media warnings have prompted public worries about health. The aim of the present study is to investigate the effects of media warnings about the possible health hazards of MP use on self-reported symptoms. Participants were 703 undergraduate students who volunteered to take part in an experimental study between August 2013 and July 2015. After completing baseline questionnaires containing information on demographics, MP usage and possible confounding variables, the participants were randomly clustered assigned to a video treatment group (watching a 5-min video about the possible health hazards of MP use) or a control group. Then, they completed another set of questionnaires containing 6 self-reported physical symptoms and the Beck Depression Inventory (BDI). Chi-squared tests, Mann-Whitney *U*-tests and logistic regression models were applied in the data analysis. Participants in the video group reported significantly more frequent headache (*P* = 0.01), fatigue (*P* = 0.00), memory loss (*P* = 0.03), inattention (*P* = 0.00), and higher level of depression (*P* = 0.05) than those in the control group. Additionally, the prevalence of memory loss (β = 0.071, *P* = 0.03) and inattention (β = 0.110, *P* = 0.00) were significantly higher in participants with higher level of depression who watched the video. Media warnings about the possible health hazards of MP use promote people to report physical symptoms and psychological problems. Considering this tendency, more moderate and scientific media information is needed to alleviate public worries about MP use.

## Introduction

At the beginning of the twenty-first century, concerns about the safety of mobile phone (MP) use, environmental pollution, food, etc. have led to a generally heightened awareness of the effects of environmental changes on health (1). Pervasive MP use is a reality of modern life in China. In 2014, there were over 1.23 billion MP users (more than 90% of the population) in China (2). The gradually increase in MP use had elevated the public's attention to the possible health effects of exposure to MP (3, 4).

People usually get information from daily media, such as internet, TV, newspapers, magazines. People would seldom search for scientific articles or reviews for health information and look into the results and discussion. These would result in the misleading of people from the media and misinterpretation of media contents. Currently, the mass media plays a vital role in informing the public about the possible health effects of exposure to MP. It is very popular to obtain health information through the Internet, with over 50% of Internet users searching for health information (5). Nowadays, mass media report health impact of MP use in a more objective way. However, information inconsistent with scientific results is still spread (6). People were more attractive to reports about possible hazardous effects of MP use. News headlines, such as “Mobile phones may cause cancer, warn world health chiefs: After years of contradictory claims, an authoritative verdict” (7) and “Mobile phones could be ‘health time bomb:' More than 200 academic studies link use with serious illnesses” (8) were published after the International Agency for Research on Cancer (IARC) issued an official report concluding that cell phone usage was “possibly carcinogenic to humans” (9). Furthermore, the report “The Inconvenient Truth about Cancer and Mobile Phones” appeared on internet in 2018 declared that “clear evidence has been found that radiation from mobile phones causes cancer” (10). These media warnings might increase people's anxiety about personal health, reinforce hypochondriasis, or cause unnecessary concern about their health status (5). Participants in one study sought to understand the interaction between the media and cancer felt that some scientific research findings were overstated by the media (11). In addition, a recent study showed that media reports about the adverse effects of Wi-Fi elevated the levels of public concern (6).

In the meantime, scientists are increasingly concerned about the potential health effects of MP use. In some epidemiological studies, headache (12–14), dizziness (12, 15), fatigue (14, 16), memory loss (17, 18), inattention (19, 20), and irritation (21, 22) have been reported as significantly related to MP use or living near MP base stations. Depression, as the most common disorder in the world (23) and a major mental problem that had been highlighted among university students (24), was also significantly associated with long-duration MP use (16, 25). Moreover, higher scores of depressive symptoms were significantly associated with more frequent physical symptoms, such as headache (26, 27), dizziness (28), and fatigue (29, 30). However, several studies reported that no such symptoms were found concerning mobile phone radiation and mobile phone use (31–33).

In the present study, we investigated the effects of media warnings about the possible health hazards of MP use on self-reported physical symptoms. Additionally, we sought to study the moderation of a psychological factor on these effects.

## Materials and Methods

### Study Design

A between-group experimental study was performed between August 2013 and July 2015 at Third Military Medical University. This trial is an analyzer- and participant-blinded trial. Participants were recruited from among the freshmen of undergraduate students and written consents were obtained before experiment. After completing the baseline survey, participants were randomly clustered assigned to either the video or control group. Participants in the video group watched a 5-min video about the health effects of MP use and completed questionnaires containing physical symptoms and the Beck Depression Inventory (BDI). Participants in the control group completed the questionnaires for physical symptoms and the BDI without watching the video.

### Ethics Statement

The protocol of the current study was approved by the Third Military Medical University Ethical Committee. All participants who participated in the study completed a written consent form before baseline survey. This study and protocol were not pre-registered.

### Subjects

A total of 703 participants were recruited from undergraduate students at Third Military Medical University. During recruitment, participants were informed that they would participate in a study on the relationship between mobile phone use and health symptoms. After obtaining written consent, the baseline survey by the questionnaires including the information on demographics, MP usage and possible confounding variables was conducted. Excluding uncompleted questionnaires, 685 (97.44%) participants were randomly clustered assigned to the video or control group. Of the received questionnaires for physical symptoms and the BDI, 674 (95.87%) valid questionnaires were used in the statistical analysis.

### Questionnaire

Baseline survey questionnaire include three parts: The demographic information, MP usage and possible confounding variables. After participants were assigned to the video group and the control group, they were asked to complete another questionnaire including self-reported physical symptoms and BDI.

The demographic information requested included name, gender (male/female), age, grade, home address, and telephone number.

MP usage included ownership of MP, the years of MP usage, daily duration of MP calls and weekly number of MP calls. The ownership of MP was assessed by the following question: “Do you own a MP?” The years of MP usage was assessed by the following question: “How many years have you used MP?” The daily duration of MP calls was assessed by the following question: “How much time do you spend making MP calls daily?” Finally, the question “How many calls do you make weekly?” was used to assess the weekly number of MP calls. For all of the above questions, MP usage included the use of other people's phones.

The self-reported physical symptoms included headache, dizziness, fatigue, memory loss, inattention and irritability, which were taken from the Zerssen complaint list (33, 34). These symptoms were assessed on a four-point Likert scale (heavy, moderate, weak, not at all). Because only few participants reported heavy and moderate symptoms, the Likert scale data were dichotomized. A symptom was considered present if it was reported with at least weak intensity (22). The Cronbach's α coefficient of the well-being items was 0.803.

Depression status during the previous week was evaluated using the 21-item version of the BDI, which is a self-report inventory created by Beck et al. (35). This inventory is a reliable and validated instrument (36), which is one of the most widely used instruments for measuring the severity of depression. Each statement in this inventory was rated from 0 to 3, yielding a maximum score of 63. The levels of depression were determined as follows: 0–9 points (minimal level of depression), 10–16 points (mild depression), and 17–63 points (severe depression) (37).

Daily physical exercise (yes/no), smoking (yes/no), and drinking (yes/no) as well as gender and age were considered to be confounding factors.

### Video

In the video group, participants watched a 5-min video, which was made by an information technology professional colleague. The title of the video was “Electromagnetic radiation—a concealed killer around us.” This video included description of electromagnetic radiation in daily life, potential health effects of electromagnetic radiation, the present status of MP use, potential cancers related to MP and some results of the studies that have investigated the possible health effects of MP use. Video was narrated in Chinese, and the English translation for the video narrator was provided as supplementary material (Supplement 1).

### Procedure

**Figure 1:**
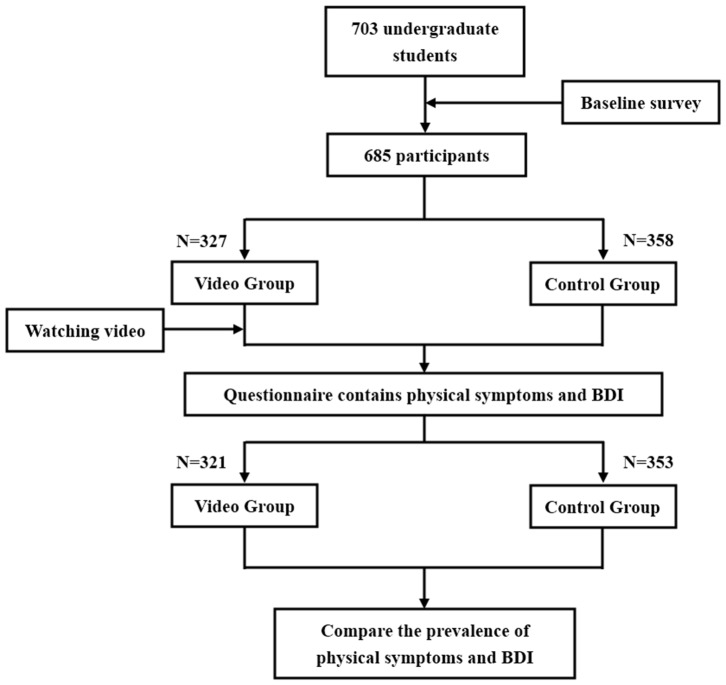
Flow diagram of trial procedure.

Before watching the video, participants provided written consent and completed the baseline questionnaires for demographics, MP usage and possible confounding factors, such as engaging in daily physical exercise, smoking, and drinking. Then, they were randomly assigned to either the video group or control group. In the video group, participants watched a 5-min video on a block of all video group participants divided into three groups in three different classrooms playing the video in the class time. Participants in control group fill in the rest questionnaire without watching the video at the same time. After watching the video, participants completed another set of the questionnaires containing 6 self-report physical symptoms and the BDI items. In the control group, participants complete these questionnaires without watching the video. The procedure was shown in Figure 1.

### Statistical Analysis

Chi-squared tests were used to compare the distribution of sex, daily physical exercise, smoking, drinking, MP ownership, prevalence of physical symptoms and the BDI score between the two groups. Student *t*-test was used to analyze normally distributed variable, such as age. Mann-Whitney *U*-tests were applied to analyze non-normally distributed variables, including years of MP usage, daily duration of MP calls, weekly number of MP calls and level of depression between the two groups. Logistic regressions were used to test whether increases in the prevalence of physical symptoms would be predicted by the higher level of depression, by watching the video or by the interaction between these two factors. The inclusion of interaction terms in the logistic regression models references the approach described by Witthöft and Rubin (10). Statistical significance was defined as *P* < 0.05 in the present study. Continuous variables were summarized using descriptive statistics [median (P25, P75)], and categorical variables were generally summarized using the corresponding percentages. Statistical analysis was undertaken using SPSS version 19.0 (SPSS Inc., Chicago, IL, USA).

## Results

Among the 703 undergraduate students recruited to participate in this study, 685 (97.44%) participants completed the baseline questionnaires and took part in the experimental portion. After the experiment, 674 (95.87%) valid questionnaires were returned and used in the statistical analysis.

### Baseline Information

The participants included 544 males (80.71%) and 130 females (19.29%). The mean age was 22.69 ± 1.76 years. Nearly every participant (98.96%) owned MP at the time of the survey, and the average length of MP usage was 5.68 ± 2.03 years. Participants spent 18.97 ± 17.15 min per day on making MP calls and made 17.49 ± 2.05 phone calls per week, on average. The detailed distribution of demographics for the two groups is shown in Table 1, and the information of MP usage for the two groups is shown in Table 2. There was no significant difference in demographic characteristics or MP usage between the two groups.

**Table 1:** Demographic characteristics for control group and video group (*n* = 674).

	**Control group (*n* = 353)**	**Video group (*n* = 321)**	***p***
**Age**			0.76^a^
Mean ± SD	22.5 ± 1.3	22.9 ± 2.1	
**Sex**			0.99^b^
Male, *n* (%)	285 (80.7)	259 (80.7)	
Female, *n* (%)	68 (19.3)	62 (19.3)	
**Daily physical exercise**			0.22^b^
Yes, *n* (%)	340 (96.3)	302 (94.1)	
No, *n* (%)	12 (3.4)	17 (5.3)	
**Smoke**			0.58^b^
Yes, *n* (%)	83 (23.5)	69 (21.5)	
No, *n* (%)	270 (76.5)	249 (77.6)	
**Drink**			0.82^b^
Yes, *n* (%)	225 (63.7)	200 (62.3)	
No, *n* (%)	128 (36.3)	118 (36.8)	

a *Student t-test was applied*.

b *Chi-square test was applied*.

**Table 2:** MP usage for control group and video group (*n* = 674).

	**Control group (*n* = 353)**	**Video group (*n* = 321)**	***P***
**Mobile phone**			
Ownership, *n* (%)	351 (99.4)	316 (98.4)	0.21^b^
Years of MP usage, median (P25, P75)	5 (4, 7)	6 (4, 7)	0.06^a^
Duration of calls/day, median (P25, P75)	10 (5, 30)	10 (8, 30)	0.26^a^
Calls/week, median (P25, P75)	14 (7, 21)	14 (7, 21)	0.89^a^

a *Mann-Whitney U-test was applied*.

b *Chi-square test was applied*.

### Effect of Watching the Video on Self-Reported Physical Symptoms

**Figure 2:**
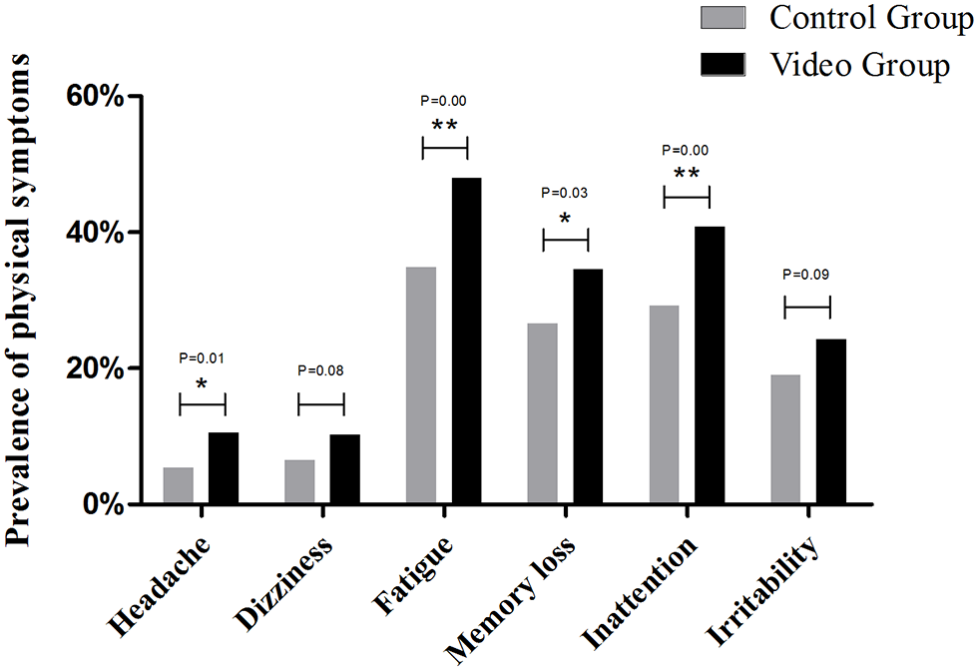
Prevalence of physical symptoms between control group and video group. *P < 0.05, **P < 0.01.

The most frequently reported physical symptom was fatigue (41.4%), followed by inattention (35.2%) and memory loss (30.9%). In the video group, all physical symptoms were more frequently reported than in the control group. Additionally, the prevalence of headache (*P* = 0.01), fatigue (*P* = 0.00), memory loss (*P* = 0.03), and inattention (*P* = 0.00) were significantly higher in the video group (Figure 2). Logistic regression analysis showed that headache, fatigue, memory loss, and inattention was significantly associated with watching video (Table 3).

**Table 3:** Association between watching video and physical symptoms (*n* = 674).

	***N* (%)**	***p***	**OR (95% CI)**	**Adjusted OR (95% CI)^#^**
**Headache**		0.01		
Control group	19 (5.4)		1.00	1.00
Video group	34 (10.6)		2.22 (1.25–3.96)	2.33 (1.13–4.80)
**Dizziness**		0.08		
Control group	23 (6.5)		1.00	1.00
Video group	33 (10.3)		1.82 (1.05–3.14)	1.49 (0.78–2.86)
**Fatigue**		0.00		
Control group	123 (34.8)		1.00	1.00
Video group	154 (48.0)		1.79 (1.32–2.45)	1.62 (1.14–2.29)
**Memory Loss**		0.03		
Control group	94 (26.6)		1.00	1.00
Video group	111 (34.6)		1.54 (1.11–2.14)	1.49 (1.02–2.15)
**Inattention**		0.00		
Control group	103 (29.2)		1.00	1.00
Video group	131 (40.8)		1.74 (1.26–2.39)	1.58 (1.10–2.27)
**Irritability**		0.09		
Control group	67 (19.0)		1.00	1.00
video group	78 (24.3)		1.42 (0.98–2.05)	1.37 (0.90–2.09)

*P Obtained through χ^2^ test*.

# *Adjusted for sex, age, physical exercise, smoke, drink, MP ownership, MP ownership, years of MP usage, minutes spent on calls daily and phone calls daily*.

*OR, odds ratio; 95% CI, 95% confidence interval*.

### Effect of Watching the Video on Depression

There was no significant difference in BDI score between the two groups. However, the level of depression of the participants in the video group was significantly higher than that of the control group (*P* = 0.05) (Table 4).

**Table 4:** Score of Beck Depression Inventory (BDI) for the two groups (*n* = 674).

	**Control group (*n* = 353)**	**Video group (*n* = 321)**	***P***
Score of BDI [median (P25, P75)]	3 (0,7)	3 (0, 8)	0.76^a^
**Degree of severity of depression**			0.05^b^
Minimal level of depression (score 0–9), *n* (%)	296 (83.9)	250 (77.9)	
Mild depression (score 10–16), *n* (%)	41 (11.6)	42 (13.1)	
Severe depression (score > 16), *n* (%)	16 (4.5)	29 (9.0)	

a *Mann-Whitney U-test was applied*.

b *Chi-square test was applied*.

### Moderation of the Effect of Watching the Video on Physical Symptoms by Depression

**Figure 3:**
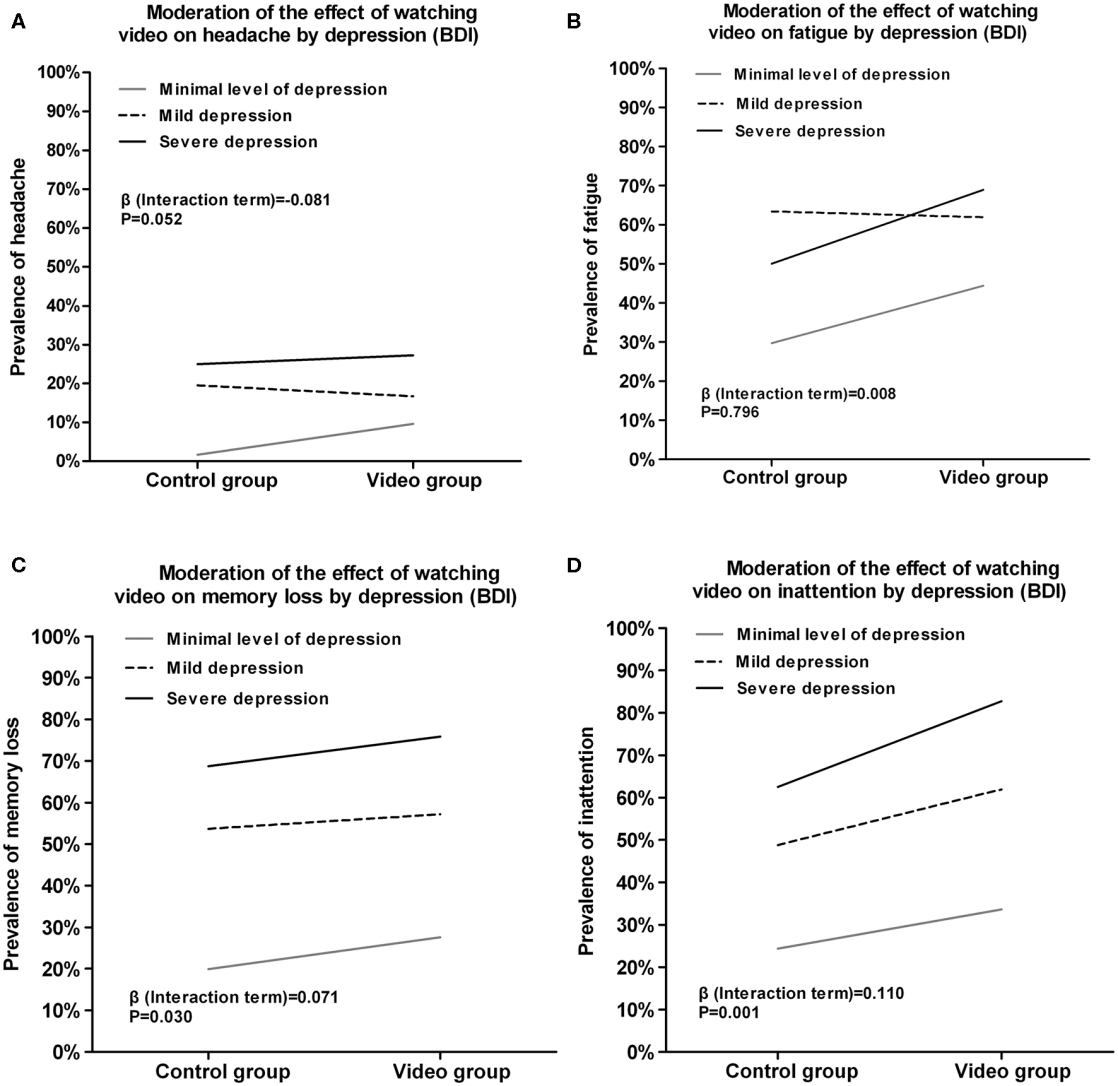
Moderation of the effect of watching video on self-reported symptoms by depression (BDI). **(A)** Moderation of the effect of watching video on headache by depression. **(B)** Moderation of the effect of watching video on fatigue by depression. **(C)** Moderation of the effect of watching video on memory loss by depression. **(D)** Moderation of the effect of watching video on inattention by depression.

To investigate the interaction effect on physical symptoms between depression and watching video, we estimated a logistic regression. The result showed that the prevalence of physical symptoms was higher among participants with higher level of depression who watched the video (Figure 3). Additionally, the logistic regressions with the prevalence of memory loss (β = 0.071, *P* = 0.03, Figure 3C) and inattention (β = 0.110, *P* = 0.00, Figure 3D) as the outcomes showed significant interactions between watching the video and depression. Repeating the logistic regressions including sex, age, daily physical exercise, smoking, drinking as confounding factors yielded similar results, and none of these confounding factors had a significant effect on the prevalence of physical symptoms.

## Discussion

As MP is integrated into our lives, more news headlines have reported the health hazards of MP use. Thus, people start to worry about whether MP use is likely to endanger their health. We investigate the effects of media warnings about the health hazards of MP use on the self-reported symptoms of young adults for the first time in China. Our results revealed that watching a video about the health effects of MP use elevated the prevalence of self-reported physical symptoms, and this effect was stronger in participants with higher level of depression.

In contrast to participants in the control group, participants who watched the video were significantly more likely to feel headache, fatigue, memory loss, inattention, and depression. Several previous studies have shown that people with long-duration MP use reported more physical symptoms (13–15) or depression (16, 25), but there was no significant difference of MP use between the two groups in the present study. Thus, we considered the effects on self-reported symptoms and depression to be due to watching the video than to MP use. It is important to keep in mind that only few participants reported heavy and moderate symptoms. Although, the frequency of symptom reporting increased in the group that watches the video, this is mostly a comparison between “not at all” with “weak” because “heavy” and “moderate” were not reported. So effects are really minor. The effects of watching a video on self-reported symptoms may be more of a nocebo effect. A television report on adverse health effects of electromagnetic field could lead participants to feel more intense tactile stimuli even in sham WiFi exposure compare to a neutral report (38). People reported sensitivity to mobile phones experienced greater depression and greater worries about general health problem which suggests that psychological factors were associated with self-reported sensitivity to mobile phones (39). Also, the effects of a 5-min video on self-report symptoms and the status of depression may be transient and without long-lasting adverse consequences. However, In this study, watching a 5-min video is a one-time trial which tries to mimic public receiving media information. In daily life, people get health information from multiple sources and for many times. The long-lasting effects of media warning on self conception should not be neglected.

Unique findings from our study include several significant interactions between watching the video and the level of depression on self-reported physical symptoms. The BDI score in this study was 4.9 ± 6.7 and 5.2 ± 6.6 in control group and in video group, respectively. This BDI score is comparable with average population, such as young male military recruits in Brazil (age: 18; BDI score: 6.93 ± 6.79) (40), non-patient adult population (age: 53.0–63.0; BDI score: 6.50 ± 6.67) (41). The results indicated that watching the video was related to the prevalence of physical symptoms, and the associations were enhanced by higher level of depression. In other words, the significantly higher prevalence of physical symptoms in the video group could also be influenced by depression level. Interestingly, frequently exposed to negative reports or affective tone in media was associated with detrimental changes in acceptors' mental health (42). When individuals obtain health-relevant information through Internet media, they might receive hints or warnings about health problems and focus too much attention on their symptoms (43) and imagine being ill, inflating their perceptions of risk (5). This could lead to rumination and increased depression symptoms (44). Additionally, that people with higher level of depression report headache (26, 27), dizziness (28), and fatigue (29, 30) significantly more frequently has also been revealed in previous studies. These results might provide support for the above finding. People reported more severe level in finishing BDI after watching the video. This might contribute to the higher prevalence of self-reported physical symptoms in the video group. Personality traits are major factors which may influence self-reported health worries triggered by the video information. The status of anxiety, somatosensory amplification, and pre-test EMF risk perceptions could guide people to report health problems in different way without regard of real feeling (6). People with idiopathic environmental intolerance attributed to electromagnetic fields (IEI-EMF) are more easily promoted to report illness when providing clue of EMF existing even there is no EMF exposure (38).

This experimental study is the first study to indicate that students who watched a video about the possible health hazards of MP use felt physical symptoms more frequently and reported higher level of depression in China. It adds evidence about the impact of the scary media effect from a Chinese student population. Based on similar previous research (6), the sample size in our study is sufficient to detect some of these outcome effects. The Zerssen complaint list assessing physical symptoms in this study had been used in previous surveys (34, 45, 46) and is suitable for individual and group settings (47). The reliability of the list has been tested for internal consistency. In this study, we used modified Zerssen complaint list. We selected six MP use related symptoms from Zerssen complaint list and translated into Chinese in order to use it in Chinese undergraduate students. The Cronbach's α coefficient of the well-being items was 0.803. However, the selection of part of complaints from this list, as well as translation into Chinese, changes the validation of the original Zerssen complaint list. Logistic regression models were repeated adjusting for various confounding factors, such as sex, age, daily physical exercise, smoking, and drinking. Therefore, we could rule out the possibility that these factors might have influenced our results. Most measures of physical symptoms and depression were based on self-reported data, which are subject to memory error and reliability concerns (48). Self-reported data could be easily influenced by personal memory, mood, and psychological stability. This could help to explicit the effect of media warning on self evaluation and self-reported symptoms.

Our results added evidence to the body of research on the effects of media warnings about the health hazards of MP use on physical and psychological health. However, several limitations should be noted. One of the main limits of this study is that most information about mobile phone use is based on self-reported information collected by questionnaire. To collect mobile phone exposure data by self-reported questionnaire is one of effective and convenient methods to collect information in epidemiological study related with mobile phone use and health which had been used before (22, 49). However, it has to be kept in mind that self-reported exposure is no valid proxy for the real exposure. To reduce the misclassification of self-reported mobile phone use, it is better to obtain detailed mobile phone use data from a mobile service supplier or use personal dosimeters to assess individual exposure to mobile phones. Secondly, using a 5-min video may not represent the real situation of people get information from mass media. The fact that watching a video in one group compared to watching nothing in the other may lead to imbalanced information in control and video group. In future study, using videos from real media material and using two videos to do the compare should be considered. Thirdly, only depression was considered as a psychological factor in the present study, we could not exclude the possibility that the effects of other psychological factors increased the prevalence of physical symptoms.

## Conclusions

In this study, based on similar mobile phone use background in two groups, watching a video about mobile phone health hazards resulted in more frequently reported health symptoms and higher level of BDI score. There is evidence that media warnings about the health hazards of MP use promote people to report physical symptoms and psychological problems in the present study. However, since few participants reported heavy and moderate symptoms, and seldom reported relatively high BDI scores, the effects of video are really minor. Greater collaboration between social media and scientific organizations is needed to provide more credible and pertinent health information about MP use.

## Data Availability Statement

The datasets generated for this study are available on request to the corresponding author.

## Ethics Statement

The studies involving human participants were reviewed and approved by Third Military Medical University Ethical Committee. The patients/participants provided their written informed consent to participate in this study.

## Author Contributions

Z-PY and LZ: conceptualization, funding acquisition, and writing—review and editing. PG, F-ZZ, and PD: formal analysis. PG, F-ZZ, M-DH, ML, and LZ: investigation. PG, F-ZZ, and LZ: methodology. PG, M-DH, and ZZ: validation. PG and F-ZZ: writing—original draft.

## Conflict of Interest

The authors declare that the research was conducted in the absence of any commercial or financial relationships that could be construed as a potential conflict of interest.
