# Supplementary material for: An Experimental Study of Effects of Media Implication on Self-Report Symptoms Related With MP Use
Source: Front Public Health. 2020 May 13;8:175. doi: 10.3389/fpubh.2020.00175 (PMC7237582; doi:10.3389/fpubh.2020.00175)
Supplement: Supplementary file 1 [file Data_Sheet_1.PDF]

Supplement 1. Translation for the video narrator.

1. Hello, everyone. Welcome to watch the science video called “Electromagnetic radiation - a concealed killer around us” made by department of occupational health, Third Military Medical University.
2. The second Industrial Revolution and the third Industrial Revolution moved forward the development of technology as well as the human society.
3. With the incorporation of modern electronic products into daily life, they bring us great convenience. In the meantime, a new problem about the health effects of electromagnetic radiation appears in our vision.
4. A lot of researches reported that the effects of environmental electromagnetic radiation were increasing daily. Previous studies found that long-term and excessive exposure to electromagnetic radiation could influence our health.
5. Electromagnetic radiation has been considered to be the fourth environmental pollution factor after waste gas, waste water and waste residue.
6. Unlike these environmental pollution factors, electromagnetic radiation is undetectable and often ignored. It must be detected by means of instruments. So the electromagnetic radiation is a concealed killer around us.
7. The electromagnetic field in our lives usually includes extremely low frequency electromagnetic field (ELF EMF) and radio frequency electromagnetic field (RF EMF).
8. ELF EMF is always produced by home appliances, electric outlets, transformers and power transmission lines.
9. The residents near these exposure to a large mount of ELF EMF. Previous research found strong evidence that children exposed to ELF EMF had a higher risk of leukemia.
10. The subsequent study found that people with long-time occupational exposure to ELF EMF have higher risk of Alzheimer's disease and breast cancer.
11. RF EMF influenced our health is always produced by mobile communication devices, such as mobile phone, computer.

12. We have a longer lifetime exposure to mobile phone (MP). When we use MP, it is close to our heads. Our heads will absorb the electromagnetic radiation produced by MP.
13. A neurologist reported that long-time MP use will cause cancer and prosecuted 8 telephone companies in 2010.
14. The new electromagnetic radiation is taken into our lives with the widely use of wireless router. When the wireless router works, it produces the electromagnetic radiation in the 2.45GHz frequency.
15. And 2.45GHz frequency is the same frequency of microwave oven. Imagine living in a big working microwave oven, what would your life be like?
16. More and more researches report that RF EMF is associated with neurodegenerative disorders, cardiovascular disorders, diabetes and cancers.
17. In May 2011, the International Agency for Research on Cancer (IARC) issued that cell phone usage was “possibly carcinogenic to humans” (2B). It is a general agreement that RF EMF will affect human health.
18. Every communications company will tell you that the electromagnetic radiation produced by their products could not exceed the limits. But there are no guarantees that they all add up is never more than the limits.
19. When we are in the office, the kitchen and the bedroom, we are always exposed to the complicated electromagnetic fields.
20. As the electromagnetic radiation is ubiquitous in our life, we would better rationally use the electronic products to reduce and mitigate the health effects of electromagnetic radiation.
